# Beliefs about and approaches to youth suicide risk screening and triage in primary care: a qualitative analysis

**DOI:** 10.1186/s12875-025-03102-7

**Published:** 2025-12-30

**Authors:** Lauren M. O’Reilly, Davis Meadors, Brigid R. Marriott, Leslie Hulvershorn, Matthew C. Aalsma

**Affiliations:** 1 Department of Psychiatry, Indiana University School of Medicine, 410 W 10th Street, Indianapolis, 46202 USA; 2 Department of Pediatrics, Indiana University School of Medicine, 410 W 10th Street, Indianapolis, 46202 USA; 3 Department of Psychiatry, Indiana University School of Medicine, 355 W 16th Street, Indianapolis, 46202 USA

**Keywords:** Suicide screening, Depression screening, Adolescent, Primary care, Risk triage, Qualitative

## Abstract

**Background:**

Nearly 9 out of 10 youth who die by suicide visit health care settings in the year prior to their death. Given the opportunity of primary care to screen for youth suicide risk, past research has focused on understanding provider attitudes toward screening and barriers to screening implementation. However, research has not robustly examined how providers utilize information gathered from screening tools. Therefore, the aim of this qualitative study was to outline existing suicide protocols and characterize attitudes and practices toward youth suicide risk screening and subsequent triage.

**Methods:**

Youth-serving primary care providers and clinic staff working at clinics in one Midwest health network participating in a randomized control trial implementing integrated behavioral health care were contacted via email by a research assistant. Nineteen individuals participated in 30-minute, semi-structured interviews regarding attitudes and practices toward youth suicide risk screening in primary care. Interviews were transcribed and analyzed using consensus deductive and inductive coding.

**Results:**

Existing clinic workflow largely pertained to screening administration. Participants discussed the intention to universally screen pediatric patients using the Patient Health Questionnaire (PHQ)-2 for sick visits, which served as gate questions for the PHQ-9. For well child visits, clinics implemented universal PHQ-9 screeners. Participants were largely not aware of standardized processes, including brief suicide risk assessment, use of consultation services, or risk triage decision-making. Interviews described themes of attitudes toward youth suicide screening, primary care protocols, risk triage decision-making, and barriers and facilitators to youth suicide risk screening. Overall, providers and clinic staff expressed overwhelming confidence in administering screens, which was considered within their scope of practice. Confidence, however, wavered when providers discussed their ability and/or comfort determining follow-up steps, providing brief interventions, and connecting youth to behavioral health services.

**Conclusions:**

Results emphasize that without adequate behavioral health assessment and intervention post-screening, primary care providers face significant burden managing suicide risk, which was often felt outside their scope of practice. Developing and adapting intervention models within primary care (e.g., integrated care models) are crucial next steps.

**Supplementary Information:**

The online version contains supplementary material available at 10.1186/s12875-025-03102-7.

## Background

In the United States (US), 42% of youth and young adults aged 10 to 24 years old who died by suicide visited a health care professional in the month prior to their death and 88% visited a health care professional in the year prior to their death. When examined by setting, youth and young adults often visited a primary care provider; specifically, 19% of those who died by suicide visited a primary care provider in the month prior to their death. Additionally, those who died by suicide were more likely to visit a primary care provider in the month and year prior to their death compared to matched controls [1]. Studies such as this have highlighted the primary care setting as a critical point of identification and intervention for youth suicide risk. This is especially relevant as high volumes of pediatric patients present to primary care. In 2023, 93% of youth presented for an annual well child visit in primary care in the prior 12 months [2]. 

Suicide prevention best practices in primary care (i.e., screening, brief assessment, brief intervention, and referral) have been outlined by national accrediting bodies and professional organizations (e.g., the American Academy of Pediatrics, US Preventive Services Task Force) [3, 4]. While prior studies have documented the implementation of such practices (e.g., suicide risk screening and clinical decision-making pathways) [5–8] and thus have demonstrated the feasibility of implementation in certain settings, the research regarding the prevalence of these services, while sparse, clearly demonstrates considerable gaps. When examining best practice uptake in primary care, past research has disproportionately focused on screening utilization and, therefore, documentation of screening gaps is most robust. As examples, a survey of medical providers in Pennsylvania found that at well child visits, 67% screened for mental health problems, 35% screened for suicide risk, 14% used a standardized tool to screen for suicide risk, and 61% screened for suicide only when indicated [9]. More recent data from a national survey of 302 pediatric and adult primary care providers found that 55% endorsed routine suicide screening (which included standardized depression and suicide-specific screening tools) and 39% endorsed targeted suicide screening [10]. Suicide prevention best practice utilization other than screening is minimally documented, yet gaps are likely prevalent. For example, one study conducting chart reviews of youth with elevated suicide risk in primary care found that 13% were asked about access to firearms, 7% were asked whether they had a safety plan, and 51% of youth were referred to behavioral health services [11]. 

In order to address suicide prevention gaps in youth primary care, research is needed to articulate the factors that may influence the uptake of suicide prevention in practice. Numerous studies have identified key facilitators and barriers to suicide prevention, again with a focus on suicide screening implementation. Barriers to screening include perceived low self-efficacy in delivering suicide interventions, uncertainty in predicting future risk, time constraints, competing demands, and concerns with clinic workflow post-screening [12–14]. Regarding facilitators, Diamond and colleagues (2012) found that endorsement of having knowledge about how to conduct a suicide risk assessment was associated with greater likelihood of reporting screening for mental health and suicide risk. Integration into electronic health records and collaboration with mental health providers have also been associated with more favorable attitudes toward suicide risk screening [12]. 

Taken together, past research has documented the difficulty of suicide prevention in primary care. However, as mentioned above, past research has predominately focused on the prevalence of youth who are screened for suicide risk or attitudes toward screening, including universal screening. Considerably less attention has been paid to how providers utilize and integrate information from a screening tool. Stated differently, research is needed on the clinical decision-making pathway after screening, including brief assessment, brief intervention, and triage. Additionally, research has minimally specified factors that may influence the use of suicide prevention for *adolescents*, which may uniquely influence provider decisions and approaches to suicide prevention compared to adults.

Research is needed to improve our understanding of current clinic suicide protocols, clinic decision making regarding suicide risk identification and triage, and attitudes toward suicide risk screenings. Doing so will promote identification of gaps in primary care suicide prevention practices for youth and implementation strategies to increase evidence-based practice uptake. The aims of this paper were to: (1) describe the existing patient workflow of youth screening and triage in a selection of primary care clinics in one health care system, and (2) identify common themes in perceptions toward and decisions made prior to and after youth screening through a thematic analysis of youth-serving providers and staff qualitative interviews.

## Methods

### Setting and participants

The current study was derived from a larger hybrid type III trial evaluating the implementation and effectiveness of an adapted pediatric integrated behavioral health program (Pediatric-Integrated Behavioral Health; Peds-IBH) aimed at increasing access to timely behavioral health care services. Note that we use the term behavioral health throughout to refer to mental health and substance use service provision broadly. Peds-IBH uses a cluster randomized, stepped-wedge design, in which 25 clinics from one large healthcare network in Indiana were randomized to three cohorts, and stepped into the study in six-month intervals. The current study recruited providers and staff from 10 clinics within the larger study from cohorts 1 and 2; one clinic was a hospital-based clinic and subject to suicide screening requirements of the Joint Commission. The clinics had completed pre-implementation needs assessments interviews with the Peds-IBH research team, had been trained on protocols for implementation, and were assigned a behavioral health provider to whom they could refer patients. Notably, goals of the Peds-IBH program were to implement universal screening and brief intervention through task-shifting and to integrate behavioral health services for children and adolescents in pediatric and family medicine clinics. Peds-IBH and the current study were approved by the first author’s Institutional Review Board (Protocol #20891).

### Data collection

We aimed to recruit approximately 20 pediatric primary care providers and staff as prior research suggests saturation (i.e., the point at which no new insights would be gathered with additional data collection) will be met at this level [15]. We targeted both providers (Doctors of Medicine or Osteopathic Medicine [MD/DO], Nurse Practitioners [NP], Physician Assistant [PA]) and clinic staff (Registered Nurse [RN], Licensed Practical Nurse [LPN], Medical Assistant [MA], administrative), as one of the aims of the current study was to understand clinic protocols in response to elevated suicide risk. We used convenience sampling; recruitment was conducted by emailing providers and staff from a list of employees provided by the clinic. Recruitment occurred in June and July 2024.

A total of 126 emails were sent to eligible participants; one email was inactive as the individual left their position after the project initiation. Of the 125 delivered emails, a total of 19 participants conducted a qualitative interview; 15 were providers (MD/DO *N* = 7; NP *N* = 3, PA *N* = 1), 4 were RNs, 3 were clinic managers, and 1 was a medical assistant. Three participants (one provider) were employed at the hospital-based clinic. Among those who expressed interest, reasons for non-participation were leaving their position (*n* = 1), scheduling difficulties (*n* = 6), and attempting to schedule an interview after qualitative saturation had been determined (*n* = 1). Refer to Table 1 for demographic information of the participants. Participants were employed in rural, suburban, and urban clinics.

**Table 1:** Demographic information of participants

	*N* (%)
Gender	
Woman	12 (63.2)
Man	5 (26.3)
Did not report	1 (5.3)
Ethnicity	
Asian	1 (5.3)
Black	1 (5.3)
Native American	1 (5.3)
White	15 (78.9)
Did not report	1 (5.3)
Ethnicity	
Hispanic	2 (10.5)
Non-Hispanic	17 (89.5)
Time in Position	
<1 year	1 (5.3)
1–5 years	8 (42.1)
5–10 years	3 (15.8)
10–15 years	1 (5.3)
>15 years	5 (26.3)
Did not report	1 (5.3)
	M (SD); Range
Age	39.9 (12.4); 24–70

All participants provided informed consent at the onset of the interview. Participants were told the purpose of the interview was to learn how their clinic responds to suicide risk among youth with the goal of developing a more robust suicide prevention infrastructure within the healthcare network. The interview guide focused on youth suicide screening, including clinic guidelines and protocols, suicide risk determination, and barriers and facilitators to youth suicide risk screening, and response to perceived risk through triage, referral, and intervention (Supplementary Material [SM] Appendix 1). Interviews lasted approximately 30 min and participants were compensated with a $30 virtual gift card. Interviews occurred in July 2024. Interviews were conducted in-person in the participant’s clinic (*n* = 4) or virtually via a secure virtual platform (*n* = 15); participants conducted interviews from the clinic or their home or car and no one else was present besides the participant and interviewer. Interviews were audio recorded and stored on a HIPAA-compliant platform. All virtual interviews were also video recorded; one virtual interview participant completed portions of the interview over audio only while driving. Given internet or cellular reception, audio recorded interviews occasionally lagged and caused gaps in the transcription.

All interviews were conducted by a masters-level trained research assistant (DM) who identifies as a man and was trained in qualitative interviews and coding during his position. Fieldnotes were not made during or after the interviews. However, initial interviews were reviewed by two doctorate-level researchers (LO and MA) to provide initial feedback, who identify as a woman and man, respectively, and collectively share 25 years of qualitative data collection analysis. Additionally, DM discussed impressions, reactions, and preliminary themes with LO throughout the process to aid in reflexibility, identification of biases, and saturation determination. No repeat interviews were conducted and transcripts were not returned to participants for feedback.

### Analysis

Video files from the interviews were transcribed with Microsoft Sharepoint software (*n* = 15); audio files from in-person interviews were transcribed manually by DM (*n* = 2) or via Microsoft Word transcription services (*n* = 2). Transcripts were deidentified and checked for accuracy by the research assistant (DM). For Aim 1, two independent coders (LO and DM) independently constructed the patient workflow upon interview review. Workflows were compared and disagreements were resolved to reach complete agreement on the presentation of the workflow. For Aim 2, transcripts were managed using NVivo Version 14. Using Thematic Analysis and a hybrid coding approach, [16] LO and DM first independently reviewed transcripts and then met to discuss a tentative codebook. From there, transcript reviews continued, and LO and DM met monthly to refine the codebook, resolve differences, and finalize themes and subthemes based on complete agreement. LO then conducted all coding within NVivo, of which 20% of transcripts (4) were reviewed by DM. No reliability coefficients were calculated in NVivo as complete agreement occurred verbally through discussion. LO coded all transcripts due to practical constraints [17]. Inductive themes were determined within deductive themes identified in the interview guide structure (e.g., protocols, risk decision-making, barriers and facilitators). Participants did not provide feedback on interpretation of results. COREQ guidelines were followed for manuscript reporting (SM Appendix 2).

## Results

### Aim 1: construction of provider workflow

The first aim of the current investigation was to determine the current provider workflow within the non-hospital-based clinics. Because the hospital-based clinic had a robust suicide protocol aligned with Joint Commission accreditation, a review of said protocol was beyond the scope of the current investigation. However, we note that the hospital-based clinic uses the Ask Suicide-Screening Questionnaire (ASQ), which is followed by the Columbia-Suicide Severity Rating Scale (C-SSRS) and Suicide Assessment Five-Step Evaluation and Triage (SAFE-T) decision-making tool.

**Fig. 1:**
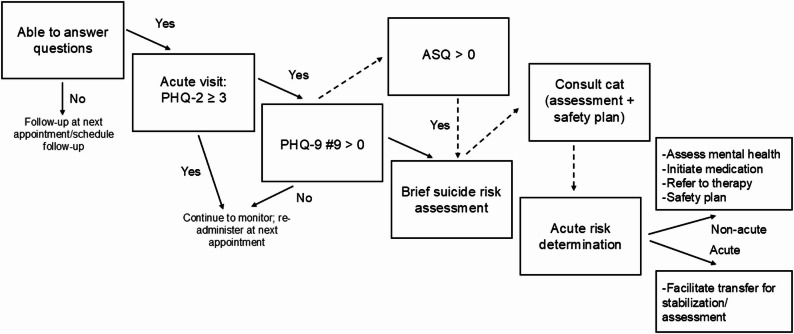
Existing pathway for non-hospital based clinics

A visual representation of the non-hospital-based clinics workflow is presented in Fig. 1. One respondent mentioned infrequent, adjunctive use of the ASQ. Clinics routinely used the Patient Health Questionnaire (PHQ), which is a depression screener than includes a ninth item indexing thoughts of death or self-harm. The PHQ-9 was implemented in annual well child visits. For sick visits, the PHQ-2 (i.e., the first two items of the PHQ-9 indexing depressive symptoms) was routinely administered, which served as a gate structure for the PHQ-9 if positive (≥ 3). Virtual, co-located psychiatric and social work consultation services [18] were a noted step either in place of or in addition to brief suicide risk assessment conducted by the primary care provider. Consultation providers were licensed mental health providers part of the health system who were virtually contacted via tablet in the primary care room with the patient. These providers routinely complete more in-depth suicide risk assessment, make triage recommendations based on imminency of suicide risk, and complete a safety plan [19].

### Aim 2: thematic analysis

Themes and subthemes are presented in Table 2.

**Table 2:** Summary of thematic codes

Theme	Subtheme
Attitudes toward youth suicide screening	Overwhelming confidence in screening
	Concern about scope of practice, especially with minimal mental health service availability
Suicide screening protocols in primary care	Minimal standard protocols for youth suicide screening
	Common, although uncertain, use of urgent consultation services
Risk triage decision making	Minimal semi-structured approaches to brief suicide assessment
	Risk triage based primarily on “active” suicidal ideation
	Differentiation between low and high risk with uncertain identification of moderate risk
	Overreliance on emergency departments to manage risk
Barriers to youth suicide screening in primary care	Insufficient time
	Perception that youth may not be truthful
	Concern about role of parents
	Conceptualizing suicide risk within depression
Facilitators to youth suicide screening in primary care	Recognition of importance of primary care role
	(Potential for) Long-standing relationships with patients
	Positive attitudes (e.g., confidence) toward screening strengthened by personal experience
	Comfort in “universal” screening messages

### Attitudes toward youth suicide screening: the roles of confidence and competency

#### Overwhelming confidence in screening

All participants expressed confidence (i.e., being “super comfortable”) in their ability to screen for youth suicide risk [Participant #6, Physician, Woman]. The experience of administering screening tools also appeared to reify screening importance, as participants recognized “[suicidality] is far more common than most people think it is” [Participant #17, Physician, Woman]. Many identified that personal comfort with screening increased due to “personal experience,” previous psychology training in school, continuing medical education or conferences, “know[ing] [they] have the resources to back [them] up to have a successful conversation,” and previous experiences where a screening tool identified a patient they otherwise would not have identified. One respondent commented on the increased comfort over repeated administration:

*I’m*,* I mean*,* at first when we started asking every patient every visit*,* I think it was a struggle just to the fact that for patients to get used to us asking the questions. But I think overall*,* I’m comfortable with asking the questions* [Participant #11, Medical Assistant, Woman].

#### Concern about scope of practice

Despite consistently documented confidence in asking screening questions around youth suicide risk, participants were clear in noting the bounds in their training, competency, and the function of primary care in screening youth but not providing treatment. Converging quotes included:

*Just picking up*,* I think our role is just picking up on*,* noticing that it’s present. Obviously*,* we can’t really treat it perfectly here*,* but we can at least refer them out to where they need to go. Therapist or psychiatrist or whatever* [Participant #14, RN, Man].

*We’re often the first person they see*,* the easiest person for them to see*,* so we definitely need to be good at screening and then referring and then just really*,* you know*,* basic things that we can do that are not outside our scope* [Participant #10, NP, Woman].

Although participants indicated comfort with screening, increasing discomfort was noted with intervention after screening (pharmacological or psychological). Regarding pharmacological interventions for cooccurring psychiatric concerns, many identified that initiating certain medications is often an immediate, accessible next step after non-high-risk suicide risk was identified as providers are “on the front line,” “folks’ insurance coverage is not great, [and the] behavioral health field is flooded and overwhelmed” [Participant #10, NP, Woman]. Participants were confident in their ability to initiate and manage antidepressants, but antipsychotic medication regimens caused considerable discomfort:

*If they don’t have just like run-of-the-mill depression*,* like if they’re schizophrenic or something*,* or if they have a really complicated bipolar with other like personality disorders. I mean*,* that’s not something that should be managed in primary care*,* but we’re kind of like limping them along until they’re able to see a psychiatric specialist* [Participant #9, PA, Woman].

Over and above pharmacological intervention, navigating clinical decision-making regarding psychological interventions appeared to be especially challenging for participations:

*Do I know how to treat depression? Do I know how to screen for suicide? Yes and yes. And you know*,* I’ll prescribe antidepressants*,* atypicals to an extent*,* but I am still a novice*,* you know*,* family practice*,* Jack of all trades*,* master of none. So no*,* I don’t know everything. I know that something is wrong and that they do need help*,* but I but maybe I don’t know the exact*,* you know*,* is it just counseling? Is it inpatient treatment? Is it outpatient treatment with counseling? Is it pharmaceutical? That that all just kind of becomes garbled up and that’s where that that person to help navigate them help out* [Participant #4, NP, Man].

Many providers commented on the broader landscape of minimal behavioral health service availability. Providers did not articulate that this discouraged their screening administration to youth. However, they did comment that identification of youth without access to timely services is ineffective and may increase burden on primary care providers, who generally feel management of behavioral health concerns (apart from certain pharmacological interventions) is outside their scope of practice. Relatedly, participants noted frequent lability in youth affect, screening score, and self-reported mental health concerns. In cases in which youth rated lowered distress since last appointment, there may be a reduced perceived need for behavioral health care; inefficient behavioral health care connection fails to capitalize on greater perceived need. One participant summarized this point and noted the ramifications that lack of timely access may have for overall system burden:

*I think that’s the most frustrating and difficult thing for us*,* current state*,* is to find access for our patients to therapy in a timely manner and then make sure that we have close follow up. So even if I can’t get a patient into outpatient therapy*,* I can certainly make a plan to see them back in my office more*,* you know*,* quickly so that I can see what’s going on*,* see what sort of things are*,* you know*,* happening. I’ve certainly seen patients where I was really concerned. And then of course*,* you’ll see them again*,* you know*,* and things*,* it’s like*,* oh*,* that was just a thing*,* I’m fine*,* you know*,* and honestly*,* I mean*,* that does*,* it doesn’t happen very often ‘cause you can usually sort through that*,* but it happens sometimes. So*,* and I think just them knowing they have at least us as a resource*,* we get used as that kind of resource more often than we*,* maybe we would like because it does sort of limit our access to our other patients. Yeah*,* but it is what it is* [Participant #3, Physician, Woman].

### Youth suicide screening protocols in primary care: consistent inconsistencies

#### Minimal standard protocols for youth suicide screening

As mentioned above, one clinic (at which three respondents were employed) was a hospital-based clinic accredited by the Joint Commission and, therefore, had a suicide prevention protocol that included ASQ administration (followed by C-SSRS SAFE-T and follow-up algorithm when indicated). Apart from those three respondents, most participants endorsed use of the PHQ-2/9 screening procedure for youth suicide risk. However, after PHQ administration, participants noted that decisions to identify adolescents at elevated suicide risk based on the PHQ results was “more…provider judgments…based on the specific situation” [Participant #2, Physician, Woman]. While there appeared to be a standardized protocol for nursing phone triage, such protocols were not adopted for providers within non-hospital-based clinics, although it was clear that providers were aware of the need to escalate to the virtual behavioral health consultation programs.

#### Common, although uncertain, use of urgent consultation services

When considering steps taken by participants after screening (again, commonly for depression and anxiety), participants discussed the use of virtual, urgent, behavioral health consultation services to complete suicide risk assessment, determine level of risk, and make treatment recommendations. Many participants relied heavily on recommendations of the behavioral health consultant and worked in collaboration to determine the necessity of emergency services. For one participant, the presence of “active” suicidal ideation prompted her to “grab the [consultation service] pretty fast to get someone that specialized on how to have those conversations” [Participant #12, NP, Woman]. However, reliance on consultant recommendations diverged for rural providers, in which emergency service transfer caused considerable burden on the patient and family:

…*when we do the [urgent consults] cart*,* if the provider on the other end is saying this person needs inpatient or this person needs to go to the hospital*,* we unfortunately my clinic’s so far out in the [region] that I am not [in the same county as the ED]. So if the patient is going to go*,* let’s say inpatient to [neighboring county emergency department]*,* I have to call 911*,* let the dispatcher know that we have a patient*,* what is going to happen?* [Participant #5, Clinic Manager, Gender Unknown].

Other participants were unclear when the urgent consultation service should be used or when it was recommended to be used. Individual decision-making about consultation service utilization was based on numerous factors, including varying degrees of participant comfort with assessing suicide risk, potential wait times to access the consultation service, the duration of the service (“hour and a half, two hours” [Participant #10, NP, Woman]) limiting room space, and prior experience in which service recommendations were not youth tailored. For example:

*We do have*,* like my providers do have some concerns about the [urgent consults] cart sometimes just because we have pediatric patients and I think a lot of them deal with like children*,* I mean*,* not children*,* adults*,* OK. So*,* but for the most part*,* it works out OK… for younger kids. I think it focuses more on planning with the kids and or planning with the kids instead of the parents because the parents are the ones who are going to be like doing the majority of the safety planning. So like the focus just needs to be tweaked a little bit* [Participant #16, Clinic Manager, Woman].

### Risk triage decision-making: “active” ideation informing dichotomous triage

#### Minimal semi-structured approach to brief assessment

Participants working in non-hospital-based settings varied in the types of questions they asked within a brief risk assessment to triage risk and no participant mentioned the use of a semi-structured brief assessment (e.g., Brief Suicide Safety Assessment) [20]. Many identified their ability to identify risk based on their “gut red flags” identified via “body language, tone, what they say” [Participant #4, NP, Man]. The brief interview often functioned as an assessment of the veracity of PHQ responses and an opportunity to identify and allay youth concerns. This was especially true when participants suspected that the youth might have reasons for answering otherwise than they felt, such as parent oversight or fear of social stigmatization or emergency department admission consequential to endorsing suicidality. Participant instinctual “gut” feelings would augment or override the youth responses on the screening measure, suggested in such convergent quotes:

*So I mean that just*,* that’s just the art of what we do*,* right? You look at the [PHQ-9] form and you’re like*,* well*,* this isn’t really true*,* right?* [Participant #3, Physician, Woman].

*The process [of determining risk] isn’t started until I talk to them* [Participant #9, Physician’s Assistant, Woman].

Participants, however, did not clarify how they made triage decisions when screening responses were not consistent with their clinical impression.

#### Risk triage based primarily on “active” suicidal ideation

While brief risk assessment varied across participants, numerous participants discussed that the presence of “active” suicidal ideation influenced their decision to assess further. Yet, providers and staff varied in their definitions of “active” suicidal ideation. For many, the presence of a suicide plan was important in their decision making, however whether “active” suicidal ideation was synonymous with suicide plan or distinct remained unclear. Other participants discussed that the recency of suicidal ideation or presence of thoughts of killing oneself (versus thoughts of death) defined “active” ideation. Note that descriptions of “active” suicidal ideation were not explicitly tied to risk triage protocols, such as the SAFE-T framework for respondents from hospital-based clinics. Examples of diverse “active” suicidal ideation descriptions included:

*I mean*,* I really go off of what they’re telling me? I mean*,* I*,* you know*,* like you said*,* yes*,* you’ve been having suicidal thoughts. Like are you having the suicidal thoughts today? Do you just*,* are you just wishing that you didn’t wake up or is it that you’re like cutting*,* you know*,* is it that you have a plan and you have access to the plan? You know*,* I kind of*,* you just have ‘cause you say*,* like*,* do you have thoughts of harming yourself or others? Like you could say yes*,* and then you could just*,* you know*,* you could be cutting*,* you know*,* which is still not good*,* but*,* you know*,* not as risky as like*,* yeah*,* I have a plan to go home and shoot myself*,* you know?* [Participant #9, Physician’s Assistant, Woman].

*I asked for specifically what are their ideas about it*,* because we’re asking if they have any suicidal ideation. So what are your thoughts about it? Tell me what that looks like for you. What kind of thoughts are you having? And then looking for are these passive or active? Do we have a plan for suicide? Does anyone know that we’re feeling like that?…Like if they tell me like my plan is*,* you know*,* going to go home and take this whole bottle of pills or I*,* I had a patient tell me I held a knife to my wrist or… trying to think*,* the other things I’ve heard. Like the more passive ones are*,* I just think it would be better off if I weren’t here*,* or I think about what if I was in a car accident and just didn’t make it out? Things like that. Not necessarily where I have a definitive plan or an idea about it.* [Participant #10, NP, Woman].

*I think it’s more of like a kid is like trying to like do something itself in that moment and parents are calling in versus if they like mentioned it like a week ago…Or like say something like I’m not*,* I don’t want to be here anymore*,* like a week ago*,* then that’s not really an active ‘cause they’re not* [Participant #16, Clinic Manager, Woman].

While participants described their process of questioning patients using a gateway question structure from thoughts of death to suicidal ideation to suicide methods and/or plan, participants also appeared to differ in what they considered to be a suicide plan. While other participants generally talked about the presence of a suicide plan, one divergent definition was as follows:

*And then*,* you know*,* if they say yes to that question [of considering actually ending your life]*,* then you’re like*,* well*,* have you thought about how you would end your life? And then*,* you know*,* obviously anybody who is actually said yes*,* that they might want to be dead is more of an active suicidal situation than someone who’s just like*,* yeah*,* I’d rather be on a beach in Aruba or sure*,* I’d trade lives with somebody if it were possible. Or*,* you know*,* I thought about just falling asleep and if I never woke up*,* that would be OK…that’s more of a kind of passive suicidal issue* [Participant #17, Physician, Woman].

Conceptualization of suicide risk varied between participants. Some categorized risk as high, medium, or low, while others used a non-high versus high binary. Several preferred not to grade risk entirely. For example, one respondent discussed his approach to identifying any risk:

*I don’t know*,* just the idea behind having a very black and white low*,* moderate high*, *really*,* really doesn’t it*,* it doesn’t mean much to me… I guess it doesn’t matter if it’s if it’s moderate or high risk. I’m going to be asking the same questions*,* you know*,* depending on what answers I get… unless you have you’re saying no to all the PHQ 9 issues. I’m going to be addressing them…So any yes is*,* is it becomes my trigger to investigate further* [Participant #4, NP, Man].

Criteria for risk categorization also differed between participants. Several of those who conceived of risk as high versus non-high risk did so based on endorsement of “active” or “passive” suicidal ideation, respectively. Conversely, others saw the presence of a suicide plan, intent to die, and/or access to lethal means as indicators of conceptualized risk:

*I don’t assign a risk*,* but first of all*,* if the patient either has a high scoring on*,* on the PHQ regardless of*,* of the answer on the suicidal question. We talk with them. I talk with them about their current mental health involvement*,* current mental health resources and then if we are talking about a positive answer towards any suicidal. Again*,* I don’t chart a low*,* medium or high risk*,* but things that would make a low risk would be lack of a plan and lack of means* [Participant #7, Physician, Man].

Participants stated numerous and various other factors they consider when conceptualizing youth suicide risk, such as PHQ score ranges, concurrent mood disorder or history of a mood disorder, “recently had any attempts,” family history, personal history of suicide attempt or ideation, recency of suicidal thoughts, and presence of family stress. Among those who endorsed use of a “moderate” risk category, moderate cases were identified as non-imminent risk with factors indicating concern for escalation or heightened urgency of need for behavioral health care. Indicators varied between participants and included PHQ-9 score, frequent passive suicidal thoughts, concerns about home and family environment, history of suicide attempt, and concurrent mood disorder. One such quote reflecting this diversity included:

*Moderate would be like*,* yes*,* I’m having suicidal thoughts. Maybe I don’t have a plan*,* but I just need help with my depression*,* you know* [Participant #9, Physician's Assistant, Woman].

#### Differentiation between low and high risk with uncertain identification of moderate risk

Moderate risk cases were often deemed likely to be responsive to outpatient therapy or to medication in cases of co-occurring mood disorders. However, participants articulated discomfort in assigning moderate risk due to resource inadequacies and delays in care. In delineating moderate versus high risk, participants converged in their concern about not addressing moderate level of suicide risk between treatment referral and treatment effect, especially due to a dearth of timely resources for youth in this category:

*You know*,* so it would still be nice to know that somebody who is*,* you know*,* kind of in that*,* you know*,* severe but not ER level. Sometimes you still don’t feel like I have the greatest place just to send them*,* you know*,* for just probably care that might be a little bit beyond me*,* but it’s also something that we can’t wait for a long period of time* [Participant 3, Physician, Woman].

While access to an urgent referral process ameliorated this concern for some participants, many saw a need to reassign moderate into either low or high risk, reinforcing the tendency toward binary risk categorization, stating:

*So there’s not many I leave in that middle ground*,* as much as it’s kind of if there is great communication at home*,* family*,* everyone feels very comfortable*,* I might kind of downgrade them to like the low risk if we’re in that medium. It’s kind of like*,* do I do more escalation or are we OK with our current plan?* [Participant 12, NP, Woman]

#### Overreliance on emergency departments to manage risk

Triage steps were, then, similarly dichotomized between emergency services and non-emergency services. Emergency services were frequently discussed as the one recommendation for those conceptualized as high risk. For example, one participant stated that if he “fear[s] a patient has a high risk of suicide, [he] always send[s] them into [the] emergency room” [Participant #8, Physician, Man]. Participants noted that relative to outpatient care, the emergency department is more efficient for youth to receive psychiatric care. Stated differently, for some physicians, the emergency department was viewed as a setting for further psychiatric evaluation and assessment, exemplified through quotes such as:

*So if we have someone who we think has an active plan for ending their lives*,* they have recently had any attempts or they certainly seem to have a very significant risk for a plan. We usually refer them directly from the office to local emergency mental health access. That’s usually part of–most of–our workflow. That allows them to access behavioral health hub*,* you know*,* for psychiatry and be assessed to see if they have risk that would put them in needing to be admitted*,* you know*,* category for those that are not that I would say the severe category* [Participant #3, Physician, Woman].

Despite the comfort and confidence that participants felt knowing youth “are getting care” when referred to the emergency department, numerous providers discussed the ethical and practical concern about the quality of care provided apart from stabilization:

*When*,* if we refer to an emergency department*,* a kid may be housed there for a significant amount of time while awaiting placement. And so they’re not really getting care. They’re in a holding pattern* [Participant #7, Physician, Man].

For patients considered non-high risk, the decision-making surrounding non-emergency services was described more inconsistently and diverged across participants. Discussed treatment options included a variety of medication initiation and management, referral to behavioral health services, and/or connection to community resources. One participant described this variability:

*We then try to sort through whether or not they would benefit from medication initiation*,* which might be in our office. It might be one*,* one of my colleagues who does*,* you know*,* medicine management*,* it may be for something more moderate*,* we might say*,* well*,* we need to make sure therapy is in place and we try to make sure we can look for those resources* [Participant #3, Physician, Woman].

One participant summarized the struggle of how to support, refer, and/or treat individuals who were not considered to be at high suicide risk in the following quote:

*As far as risk detection and assessing*,* I feel like we’re we have pretty*,* pretty solid things in place. It’s not*,* it’s not necessarily assessing it*,* it’s the following up…What comes next? We have like an appetizer menu and we need entrées* [Participant #4, NP, Man].

### Barriers to youth suicide screening: time, trust, and caregivers

#### Insufficient time

Numerous participants noted the lack of sufficient time to respond to a positive depression screen (non-zero response to the ninth item on the PHQ-9). Difficulty to “squeak in an extra 30 minutes” [Participant #2, Physician, Man] is further challenged by other presenting concerns that the caregivers raise and medical tests that need to be completed. While participants recognized their obligation to “give a patient like that all the time in the world no matter how many patients behind” [Participant #4, NP, Man], it was not without an emotional and cognitive toll moving quickly to another patient and considering “patient dissatisfaction” [Participant #7, Physician, Man]. Quotes summarizing the incompatibility between time pressure and addressing suicide risk included:

*You know*,* it is always a bit of a disaster in the sense that if someone is actively suicidal*,* even passively suicidal*,* you know*,* OK*,* they come in for a 20 min urgent care appointment for mental health and they’re going to be in your clinic for an hour to an hour and a half probably. You know*,* you will have to spend two to three times longer with them and then*,* you know*,* roll in the iPad*,* have the behavioral health team evaluate.*

*I have two patient rooms. Usually they’ll find me another room if I have a patient who’s going to be in the room for a very*,* very long time. So*,* you know*,* trying to put other patients someplace else. But yeah*,* I mean it completely–it will wreck it will wreck your schedule for the day*,* you know*,* and then it’s exhausting.*

*It’s emotionally exhausting. So trying to move on to the next patient and and deal with them appropriately can*,* can be a challenge* [Participant #17, Physician, Woman].

Participants also described the need to schedule follow-up visits if the suicide risk was determined as not requiring immediate attention. Scheduling follow-up appointments, in turn, created the potential consequence of youth falling through the cracks:

*But the ones that just screen high for depression*,* I feel like it’s again*,* and you know*,* I noticed this is high. Let’s work on this but come back in a week to talk more. And again*,* with families*,* with work*,* everything*,* it’s hard to usually get those kids back in* [Participant #12, NP, Woman].

#### Perception that youth may not be truthful

In addition to the time constraint barriers, participants commonly brought up the concern that they were concerned about or perceived youth to not be truthful in their responses to screening questions. For participants, a false negative could be caused by depressive symptoms and/or “active” ideation hindering the ability to “express themselves” or not wanting to tell a provider, as well as the presence of caregivers in the room. One participant provided the example of how depression may affect responses through survey fatigue:

*Maybe they’re so tired and exhausted from depression*,* they don’t even want to give you the time to answer these questions truthfully. They just think they’re smart enough to move on* [Participant #4, NP, Man].

#### Concern about role of parents

Accounting for the role of the caregiver was a complex consideration for many participants, as the presence of a caregiver was perceived as decreasing the likelihood of a youth discussing suicidal ideation. Caregivers may also want to discuss a chief complaint other than behavioral health concerns and/or suicide risk, which may be in conflict with youths’ priorities or the necessity of following up with positive screens. Participants discussed how in many instances, caregivers often complete the screener on behalf of their youth or refuse to complete the screen:

*Some parents don’t like for their kids to fill [depression screeners] out*,* which is totally their decision*,* their right*,* but I think that could also be something that I’m not sure if maybe we need to educate them on why it’s important for us to do this. But like I said*,* it is their child and it’s their decision to refuse. It is their right to refuse. So that could be also something as well*,* but I don’t think that could be prevented unless we do further education* [Participant #13, RN, Woman].

#### Conceptualizing suicide risk within depression

A common subtheme identified was the interchange between depression and suicidality. Many participants often answered questions pertaining to depression and not suicide. While interview questions were specific to suicide, depression and suicide were often synonymous for participants, with such examples:

*I don’t know if there is a flat protocol for elevated risk of depression*,* suicide* [Participant #4, NP, Man].

*If I have someone that I would consider a low to moderate risk depression patient and then I refer to a psychiatrist*,* man*,* we’re talking like six month wait like typically anywhere. Now if it’s high and I make a phone call*,* yes*,* I can jump the line*,* as you should*,* but that’s what happens–Your low to moderate gets pushed back*,* gets pushed back* [Participant #4, NP, Man].

Relatedly, one provider commented that the decision to administer a suicide screen was a moot point as youth receive an annual PHQ-9:

*Right*,* yeah we don’t*,* yeah*,* we don’t [determine when to screen for suicide risk]*,* we just*,* we don’t try to assess risk*,* we just have the questionnaire [PHQ-9] to all the patients in that category* [Participant #3, Physician, Man].

### Facilitators to youth suicide screening: the unique role of a primary care provider

#### Recognition of importance of primary care role

Participants identified the importance of screening specifically within primary care settings, noting that primary care is well-suited to detect emerging risk and connect youth to preventative services as primary care is “able to catch [risk] before it gets to that point” of being more severe [Participant #9, Physician’s Assistant, Woman]. One participant, in fact, believed it be a duty of primary care to initiate conversations around suicide risk.

#### Potential long-standing relationship with patients

Participants frequently identified the unique role of pediatricians to establish long-standing continuous care for youth, thus allowing for the development of a trusting relationship. One participant explained this relationship:

*If we have some continuity of care*,* especially in Pediatrics as a pediatrician*,* somebody who has known a child for a while*,* there’s usually some level of trust there. So frequently the pediatrician is pretty important in terms of being able to identify and being someone that*,* you know*,* a child can confide in when they’re having mental health issues* [Participant #17, Physician, Woman].

#### Positive attitudes toward screening strengthened by personal experience

Four participants shared their lived experience with suicidality, whom all discussed their strong belief in the importance of screening for suicide risk. For example:

*One of the reasons I think I’m more comfortable*,* it’s just because I have in*,* you know*,* in my personal life*,* I’ve had someone commit suicide. So I’m pretty*,* you know*,* passionate about the screening and understand personally how that affects the people left behind* [Participant #6, Physician, Woman].

#### Comfort in “universal” screening messages

Many participants identified that communicating to patients that screening is universal eased their own concerns about screening patients. Therefore, if patients were confused by questions regarding depression, participants explained that all patients are asked these questions. Participants noted that these universal depression screening approaches, in turn, appeared to change the culture around behavioral health, depression, and suicide risk screening broadly:

*I think it creates more of an open environment where it doesn’t feel as bad to talk about. You know*,* in the past*,* I feel like we sheltered those thoughts*,* where now*,* I feel like our clinic does a very great job of like*,* if you have these thoughts*,* that’s OK. We just want to be here to help and kind of work through them* [Participants #12, NP, Woman].

## Discussion

The aims of the current investigation were to elucidate perceptions of the existing provider workflows regarding suicide risk screening for youth presenting to primary care in one health care network (Aim 1), as well as provider and staff attitudes and approaches toward suicide risk screening more generally (Aim 2). Regarding Aim 1, the workflow identified was not dissimilar to primary care workflows utilized in other health care networks in which the PHQ-9 (or a modified youth version) is often administered to youth, which then serves as a gateway for further follow-up suicide assessment. For example, Crandal and colleagues (2022) outlined the screening workflow in Rady Children’s Hospital in San Diego, including primary care clinics. Universal depression screening using the PHQ-9 was implemented; if youth endorsed moderate/severe PHQ-9 scores of “yes” to item 9, they were given the C-SSRS. If any items were endorsed on the C-SSRS, then “safety precautions” were initiated, which may have included consulting mental health providers and conducting a safety plan [21]. Alternatively, the Children’s Hospital of Philadelphia’s primary care clinics universally administered a modified PHQ-9 including items of thoughts of death, suicidal ideation, and lifetime suicide attempt. If youth endorsed any of the three items, primary care providers asked about life worth living and plan, which then served as a gate to past-week desire to die, suicide plan, and suicide attempt. Authors stated that positive endorsement then prompted “suicide crisis resources and emergency department intake process.” Otherwise, the provider referred the patient to behavioral health services [12]. 

The workflow identified in the current study differed in the following ways. While positive endorsement of the PHQ-9 ninth item prompted risk screening questions and/or brief risk assessment (conducted by the provider or urgent consult services) for all patients, the participants did not indicate their knowledge in standardization in *how* to conduct such follow-up questions/assessment (e.g., specific questions). Triage decisions based on identified risk were not standardized. It was unclear when consultation services would be utilized, when a referral to behavioral health would be placed, and/or brief interventions would be provided. The one exception is the hospital-based clinic regarding screening, assessment, and triage referral processes, which included the administration of the ASQ followed by the C-SSRS SAFE-T. The findings highlight that screening implementation may only be the tip of the iceberg when considering the implementation of suicide prevention in primary care. Considerable gaps exist in our understanding of factors affecting the implementation of a provider workflow throughout the screening process leading to brief assessment and intervention. As an example, Horowitz and colleagues (2022) conducted a quality improvement project and implemented the ASQ in pediatric primary care settings. They then subsequently developed a suicide screening workflow found in the National Institutes of Mental Health ASQ Toolkit (www.nimh.nih.gov/ASQ). While provider, patient, and parent feedback were generally positive regarding screening implementation, it is unclear which implementation strategies are needed to implement a comparable workflow in different primary care clinics. The authors identified initial training and workflow development were likely critical in supporting screening youth. Provider training is an area of ongoing research development, [22] yet future research is needed to examine which implementation strategies are needed in diverse primary care settings to promote protocol adoption in other settings.

Regarding Aim 2, qualitative interviews identified the following findings. Providers and staff were confident in their own ability or the ability of the providers, respectively, to screen for suicide risk. Importantly, the interview question was specific to screening not intervention. While not synonymous, providers considered PHQ-9 screening to be synonymous with suicide risk screening and providers commonly administered and interpreted PHQ-9 #9 scores as indicators of suicide risk. Therefore, it is unsurprising that they were confident in their ability to administer “suicide” screens when asked. Confidence in screening, despite lack of endorsement of adequate training in suicide risk assessment, is consistent with past research. Only 25% and 33% of primary care providers reported having adequate training or knowledge in suicide risk assessment, respectively, and 64% reported being comfortable talking about suicide [9]. A prominent subtheme was the role of lived experience with suicide risk, whether personally or professionally. Individuals who discussed how their lives have been affected by suicide were more fervent in their language regarding support for the role of primary care in suicide risk screening. This is consistent with prior research suggesting that having had a patient with suicide risk in the past year was associated with increased odds of screening at well child and sick visits [9]. Research has been devoted to disclosure of mental health and suicidal thoughts/behaviors in the workplace; [23] however, we are unaware of research examining how providers or staff sharing lived experience with suicide (or broader anti-stigma campaigns) may facilitate suicide prevention implementation in primary care.

Hesitancy was detected when providers discussed the difficulty of determining how to care for a youth with elevated suicide risk, especially when behavioral health services were lacking. Providers identified the increased burden on primary care to manage suicide risk and feeling ill-equipped to do so. Poor accessibility and/or availability to behavioral health care has been identified in prior studies as barriers to suicide risk screening in primary care [24, 25]. Providers articulated in the current study that while screening was viewed within their scope of practice, suicide intervention was not. This finding is consistent with a recent qualitative study of 18 pediatric primary care providers who discussed their hesitancy managing suicide risk [26]. The findings speak to the tenuous nature of implementation suicide prevention protocols without adequate supports for providers and mental health treatment options. Suicide prevention implementation in one healthcare network shows promise in increasing rates of safety planning,^27^ yet more research is needed to elucidate how to implement robust suicide prevention and which implementation strategies are most effective.

One emergent qualitative theme that we are unaware of in prior research was the discussion of triaging into two groups with minimal and inconsistent conceptualization of moderate risk, which was then related to dichotomized triage options. Providers were often, understandably, concerned with identifying high, acute risk, which was often determined by the presence of “active” suicidal ideation. Providers varied in their definition of “active” suicidal ideation, but it mostly included the presence of a suicide plan. Similarly, the decision to initiate emergency services was often discussed by providers. Options for youth with elevated, non-imminent suicide risk were minimal and providers did not have protocols to aid in decision-making regarding non-imminent suicide risk (excluding the SAFE-T decision-making tool in the hospital-based clinic). This clear gap in determining risk categorization (and how to tailor such categorization for primary care providers) is a significant addition to the field.

The role of primary care providers in monitoring and addressing youth behavioral health concerns, including suicide risk (e.g., initiating and managing psychiatric medication, providing brief interventions), is controversial as provider comfort and beliefs about scope of practice appear to be incompatible with the reality of behavioral health provider shortage and accessibility of pediatricians. DeCrane and colleagues (2024) found through a survey of 102 parents that pediatricians were the most likely resource parents would contact if their child was suicidal after behavioral health care professionals [28]. Integrated primary care models, which are broadly defined as services that combine physical and behavioral health services often via primary care, offer a solution to reduce demands on primary care providers [29]. The ability to consult with an urgent behavioral health team was favorably discussed by participants as a needed option; however, it was uncertain when providers used consultation services and how consultation recommendations were integrated into practice. Prior research has supported the integration of behavioral health into primary care [13]. Results from a cluster randomized implementation trial demonstrated that implementation of a suicide protocol (i.e., clear screening practices, same-day connection with licensed social workers to conduct safety plans) was associated with a reduction in suicide attempts after primary care visits compared to usual care among adult patients [27]. Additionally, results from a mixed-methods implementation study of a suicide prevention protocol among primary care adult smoking cessation programs identified that team-based approaches was perceived as an important site-level characteristic for protocol uptake [25]. Timely access to behavioral health care is likely crucial in suicide prevention protocols; however, research is needed to understand the clinic context that may support or interfere with integrated care, youth and family attitudes toward consultation models and integrated care, and when providers consult with and/or refer to behavioral health care professionals. While integrated care may be the optimal solution, it is not universally implemented, and primary care providers are still faced with the challenging reality of managing suicide risk without adequate time, training, or reimbursement models for behavioral health [30]. Therefore, research is needed on training models to determine amount of suicide prevention training and frequency of training [22] for sustained usage, as well as potential hub-and-spoke model training models (e.g., Project ECHO) [31]. 

The accessibility of pediatricians and family medicine doctors also interacts with race and ethnicity. Prior research examining the density of primary care providers and likelihood of receiving a behavioral health screen found that Black and Hispanic youth were more likely to be impacted by greater physical distance to a primary care provider than White youth [32]. Stated differently, while greater distance to a primary care provider was associated with reduced likelihood of being screened for behavioral health concerns, this association was more pronounced for Black and Hispanic youth. Therefore, access to primary care providers, and lack thereof, is particularly impactful to minoritized youth. Equipping primary care providers with the knowledge, skills, and confidence to screen and address suicide risk is a matter of equity. Importantly, however, the perspectives and experiences of youth of color have been largely absent from research informing screening practices and highlights an important future research direction.

As noted in Aim 1, there was minimal discussion of standardization of post-screening suicide protocols within and across clinics apart from universal depression screening over age 12 at annual well child visits. Providers discussed their use of questions consistent with items found on standardized screeners (e.g., presence of past/recent suicidal ideation, suicide plan, access to lethal means), but providers did not indicate routine administration of these questions. Rather, providers identified “gut” intuition or prior knowledge about a patient to determine risk. This is consistent with prior qualitative research with primary care providers using “gut feeling” or instinct to determine when to assess further [24]. While one hospital-based clinic in the current study employed a standardized protocol, research is needed on providers’ knowledge of the protocol, as only three respondents (one provider) participated in the current study. Of note, the healthcare system did mandate video-based training on the use of the CSSRS SAFE-T; however, respondents did not appear to have retained information from that training, if they completed it. Even in the place of standardized protocols, provider knowledge may atrophy, warranting future investigation.

Given that the majority of youth are not at risk for immediate suicide intervention,^12^ the interviews highlighted the considerable gap in decision-making supports for providers, as well as brief interventions for youth at and shortly after their primary care visit. Prior studies have documented that among youth who endorse thoughts of death, lifetime suicide attempt, or past-month suicidal ideation, only 50% were referred to behavioral health, 46% received psychotherapy, and 19% received antidepressant medication [12]. Future research would benefit from further tracking consultation rates, referral rates, treatment initiation, and treatment engagement rates, as well as investigating the implementation of evidence-based suicide interventions for youth identified in primary care at non-imminent risk. These may be standalone services (e.g., self-guided, mobile app platform interventions) and/or may bridge care while youth remain on long waiting lists for behavioral health services. Relatedly, research on patient matching to appropriate interventions (e.g., based on severity) is critically needed.

### Clinical implications

While the current study is limited to a small sample of providers in one healthcare network, we propose a set of clinical recommendations based on the findings. We first emphasize that the standardization of protocols beyond screening measures is paramount, including when to use consultation services, when to refer to behavioral health services, and when to conduct brief interventions (e.g., safety plans). Second, training and offering ongoing support for providers on how to conduct brief assessments is needed. While risk determination is a nuanced clinical process that often involves the consideration of numerous points of information (including from caregivers), we would anticipate that training providers on how to efficiently conduct risk assessments would increase confidence to implement screening, ease the burden on consultation services (if existing), and reduce unnecessary recommendations to the emergency department for suicide risk assessments. The latter point is significant, as use of emergency departments to conduct risk assessment and triage care may result in high financial burden on families and overwhelm a taxed system with limited bed placements [33]. Third, integrated care models offer significant promise to conduct ongoing risk assessments and provide treatment. Many clinics, however, may face barriers in the implementation of integrated care, further emphasizing the need for primary care providers to be well-trained in brief suicide risk assessment and suicide-focused brief interventions (e.g., safety planning, lethal means counseling). We recommend the Suicide Prevention Resource Center (https://sprc.org/online-library/suicide-prevention-toolkit-for-primary-care-practices/) as a resource to consult when implementing suicide prevention in primary care clinics. We note that the exploration of and advocacy for sustainable billing practices to support mental health staff and screening practices for primary care providers continues to be vital. Examples may include task-shifting and use of community health worker billing codes [34]. 

### Strengths and limitations

This study was strengthened by the inclusion of clinic staff as compared to only examining provider perspectives. As noted in the introduction, while studies have implemented suicide screening protocols, past research has predominantly focused on attitudes toward suicide screening, rather than exploration into the decision-making process after the administration of a screening tool. The current study’s investigation into how providers utilize screening information is an addition to the field. The results should be considered within the context of several limitations. First, the interviews were captured from providers and staff within one health care network in the Midwest, which may limit generalizability to other health care networks in other geographic locations. Second, we had a small sample size of participants (*n* = 19); however, we conducted qualitative interviews until saturation was met. Third, participant demographics were predominantly White, Non-Hispanic, and female-identifying individuals. Diverse views are needed to consider how providers conceptualize and triage risk, especially given the diversity of youth presenting to primary care compared to behavioral health care. Fourth, recruitment was conducted via convenience sampling. This may have biased the sample to individuals who were interested in discussing suicide screening and triage, potentially presenting more favorable views regarding the topic. Fifth, details about PHQ-9 administration were not included in the interview guide, which would have provided a more nuanced understanding of suicide screening in clinics. As examples, clinics varied in modality of administration (verbal vs. self-administration) and clinic procedures for screening in other languages than English. Finally, the interview guide did not probe for beliefs about organization- (e.g., leadership) or policy-level factors that may hinder or facilitate youth suicide prevention within primary care. Investigation of such implementation factors at these levels is greatly lacking.

## Conclusion

The current study investigated the current patient workflow across 10 primary care clinics serving youth populations, as well as examined attitudes toward youth suicide risk screening and clinical decision-making processes after screening. The findings highlight minimal standardization in suicide protocols for non-hospital-based clinics, apart from universal depression screening administration. Thematic analysis indicated confidence in screening with hesitancy toward scope of practice post-screening, The findings emphasize the need for implementation research to examine suicide protocols/workflow implementation, as well as examine brief interventions for youth who are not considered to be at imminent/acute suicide risk.

## Supplementary Information

Supplementary Material 1.

## Data Availability

The dataset supporting the conclusions of this article are not publicly available. Data may be made available upon reasonable request.

## Abbreviations

US: United States
Peds-IBH: Pediatric Integrated Behavioral Health
HIPAA: Health Insurance Portability and Accountability Act
COREQ: Consolidated Criteria for Reporting Qualitative Research
NP: Nurse Practitioner
RN: Registered Nurse
PHQ-2: Patient Health Questionnaire-2
PHQ-9: Patient Health Questionnaire-9
ASQ: Ask Suicide-Screening Questions
C-SSRS: Columbia Suicide-Severity Rating Scale
