# Supplementary material for: Beliefs about and approaches to youth suicide risk screening and triage in primary care: a qualitative analysis
Source: BMC Prim Care. 2025 Dec 30;26:415. doi: 10.1186/s12875-025-03102-7 (PMC12755024; doi:10.1186/s12875-025-03102-7)
Supplement: Supplementary file 1 — Supplementary Material 1. [file 12875_2025_3102_MOESM1_ESM.docx]

**Appendix 1**. Semi-structured Interview Guide.

We are interested in studying how to identify youth at risk for suicide in various settings, such as primary care. Our goal is to learn more about how your clinic responds to suicidality and suicide risk among youth to ultimately help inform robust suicide prevention infrastructures within multiple systems of care.

- 1. Report demographics
     1. Age, gender, race, ethnicity
     2. Position title
     3. Time in position
  2. What do you think are the causes of suicide risk in youth?
  3. How does your clinic screen for youth suicide risk?
  4. How is it determined *when* to screen for risk?
  5. Are you involved in screening?
     1. If so, how do you determine when someone is at low risk? Moderate risk? High risk? How do those risk categories inform your decision making?
     2. If not, who makes decisions around risk? Who further evaluates risk when youth are screened?
  6. If someone is identified as being at elevated suicide risk, what are the procedures/protocols in place? When might there be deviations from those procedures?
  7. What are your beliefs about conducting suicide risk screening?
  8. What hesitancies do you face when conducting suicide risk screening (if you do or if you were asked to)?
  9. What challenges do you encounter or foresee when screening for suicide risk in youth?
  10. Has anything in particular, whether clinical interactions, comments from other providers, etc., that has changed your perspective on suicide screenings?
  11. How comfortable do you feel implementing suicide screens? Why?
      1. What would make you feel more comfortable/confident conducting suicide risk screenings?
  12. How would you like your clinic to detect and respond to suicide risk? What do you think is done well? What can be improved?
      1. What supports do you need when it comes to suicide risk detection and intervention?
  13. Once a youth is detected at being at elevated risk for suicide, are there brief interventions you or the clinic implement (e.g., planning for safety, talking about access to lethal means, talking to parents)?
      1. If so, how confident do you feel providing brief interventions?
  14. How feasible is it to implement brief interventions for suicide risk in primary care?
      1. How does capacity affect feasibility?
      2. What may facilitate brief interventions? What may get in the way of implementing brief interventions in your clinic?
  15. Do you think your clinic would be interested in implementing brief interventions? Or receive training on brief interventions? Why?
  16. If a youth is considered at immediate suicide risk and is sent to the emergency department, how would you and the clinic like to follow-up with youth?
  17. Is there anything I haven’t asked about that you’d like to share in your experience with interacting with suicidal youth?

**Appendix 2.** COREQ checklist.

Refer to attached document.
